# Differentiation Between G1 and G2/G3 Phyllodes Tumors of Breast Using Mammography and Mammographic Texture Analysis

**DOI:** 10.3389/fonc.2019.00433

**Published:** 2019-05-29

**Authors:** Wen Jing Cui, Cheng Wang, Ling Jia, Shuai Ren, Shao Feng Duan, Can Cui, Xiao Chen, Zhong Qiu Wang

**Affiliations:** ^1^Department of Radiology, Affiliated Hospital of Nanjing University of Chinese Medicine, Nanjing, China; ^2^Department of Graduate, Bengbu Medical College, Bengbu, China; ^3^Sir Run Run Hospital, Nanjing Medical University, Nanjing, China; ^4^GE Healthcare China, Shanghai, China

**Keywords:** phyllodes tumors, classification, mammography, artificial intelligence, machine learning

## Abstract

**Purpose:** To determine the potential of mammography (MG) and mammographic texture analysis in differentiation between Grade 1 (G1) and Grade 2/ Grade 3 (G2/G3) phyllodes tumors (PTs) of breast.

**Materials and methods:** A total of 80 female patients with histologically proven PTs were included in this study. 45 subjects who underwent pretreatment MG from 2010 to 2017 were retrospectively analyzed, including 14 PTs G1 and 31 PTs G2/G3. Tumor size, shape, margin, density, homogeneity, presence of fat, or calcifications, a halo-sign as well as some indirect manifestations were evaluated. Texture analysis features were performed using commercial software. Receiver operating characteristic curve (ROC) was used to determine the sensitivity and specificity of prediction.

**Results:** G2/G3 PTs showed a larger size (>4.0 cm) compared to PTs G1 (64.52 vs. 28.57%, *p* = 0.025). A strong lobulation or multinodular confluent was more common in G2/G3 PTs compared to PTs G1 (64.52 vs. 14.29%, *p* = 0.004). Significant differences were also observed in tumors' growth speed and clinical manifestations (*p* = 0.007, 0.022, respectively). Ten texture features showed significant differences between the two groups (*p* < 0.05), Correlation_AllDirection_offset7_SD and ClusterProminence_AllDirection_offset7_SD were independent risk factors. The area under the curve (AUC) of imaging-based diagnosis, texture analysis-based diagnosis and the combination of the two approaches were 0.805, 0.730, and 0.843 (90.3% sensitivity and 85.7% specificity).

**Conclusions:** Texture analysis has great potential to improve the diagnostic efficacy of MG in differentiating PTs G1 from PTs G2/G3.

## Introduction

Phyllodes tumors (PTs) are rare breast fibroepithelial neoplasms that account for <1% (1, 2) of all breast tumors and 2–3% of all fibroepithelial breast lesions (3, 4). PTs was originally described in 1838 as “cystosarcoma phyllodes” because of their leaf like pattern of growth and internal cystic degeneration. PTs usually showed benign biological manifestations. However, approximately 20–30% of resected PTs are malignant and approximately 25% of malignant ones show metastatic features (5). A prominent and widely accepted grading system has been reported by the World Health Organization (WHO) 3-tiered classification. PTs are classified as benign, borderline, and malignant based on the semi-quantitative evaluation of key histologic findings, which include stromal cellularity, stromal atypia, stromal mitosis, and stromal overgrowth (6).

PTs may occur in any age group from adolescents to the elderly but most commonly in women aged between 35 and 55 years (1, 4). Surgical resection is the fundamental treatment for PTs. However, surgical approaches are generally selected based on the histologic grade. Wide excision or mastectomy is usually performed in PTs Grade2 (G2)/G3 (7–9). Therefore, the preoperative differentiation between PTs G1 and G2/G3 would be especially useful for surgery planning. Fine-needle biopsy is considered to be a highly accurate technique in PTs diagnosis. However, it is not proper to be used for PTs grading because of inadequate cytologic samples and the heterogeneous nature of the tissue composition in PTs (10, 11).

Various radiologic methods, including mammography (MG), ultrasound (US), and magnetic resonance imaging (MRI) have been used to preoperatively grade PTs (12). The MG and US showed limited potential in predicating PTs grades. MRI may be a useful imaging approach. However, some patients cannot undergo MRI examination because of biomedical metal stents or contraceptive ring implantations, which is very common among Chinese women. In addition, MRI examination is expensive and time consuming. Therefore, surgeons prefer direct operation after receiving US and MG examinations. It would be valuable to find a way to improve the diagnostic performance of MG or US.

Recently, artificial intelligent (AI) technology and radiomics, computer-aided texture analysis has been used for diagnosis, treatment response and prognosis evaluation in cancer patients. However, few studies have used the method of mammography combined with mammographic texture analysis to grade the PTs up to now. The purpose of this study was to determine the diagnostic performance of mammography and mammographic texture analysis in the differentiation between G1 and G2/G3 PTs.

## Materials and Methods

The Declaration of Helsinki was adhered to throughout the entire study. The protocol was approved by the Institutional Review Board of the Affiliated Hospital of Nanjing University of Chinese Medicine. The need for informed consent was waived by the Institutional Review Board, due to the nature of this retrospective study.

### Patients

**Figure 1:**
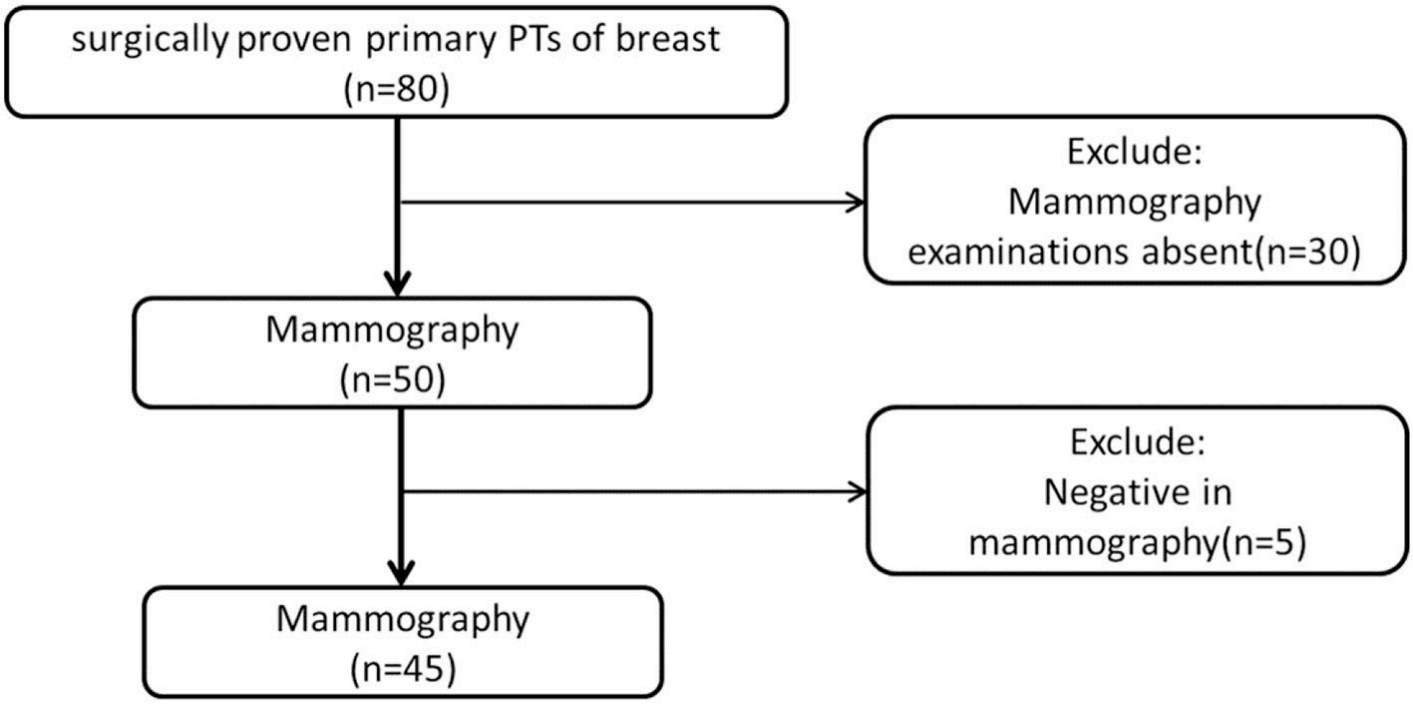
Flow diagram of patients' selection.

From February 2010 to October 2017, we obtained data from 80 female patients with surgically proven primary PTs, from our data warehouse. The patients' ages ranged from 25 to 70 years old (mean 46.58 ± 9.54). The inclusion criteria were as follows: (1) patients with surgically proven primary PTs; (2) patients who did not undergo any treatment before surgery; (3) patients who underwent preoperative mammography; (4) with a visible lesion on the mammography images. Finally, 35 cases were excluded due to the absence of MG examination (*n* = 30) or negative MG findings (*n* = 5). A total of 45 patients were included in this study (Figure 1). According to the WHO 2012 classification for PTs, the PTs were divided into G1, G2, and G3 in this study. We obtained information about the tumors growth speed by tracking the patient's previous images (including mammography, ultrasound, and MRI) or by asking about the patients feelings. A tumors diameter doubling within half a year is defined as a rapid growth tumor, while the remaining is defined as a slow growth tumor. Tactility was defined as hard like the forehead, medium like the nose and soft like the lips.

### Mammography Examinations and Images Analysis

Bilateral digital MG examinations were performed using the GIOTTOIMAGE 3D (IMS, Bologna, ITA), and choosing fully automatic exposure control mode, including the routine craniocaudal (CC), and mediolateral oblique (MLO) views. The dicom images were obtained from the Picture Archiving and Communication Systems (PACS). Two radiologists (>8 years' experience in mammography), who were blinded to pathological findings, analyzed the images. The following imaging information was evaluated: tumor size, margin (well- defined or ill-defined border), shape (oval, weak lobulation, and strong lobulation /multinodular confluent), density (hypodensity, isodensity, or hyperdensity), homogeneity (yes or no), the presence of fat or calcifications, and the presence of a halo-sign (a low density fat ring caused by the tumor pushing against surrounding structures). In addition, some indirect manifestations, including breast composition categories of American College of Radiology (ACR), skin thickening, venectasia, and axillary lymphadenectasis (the short diameter >1 cm) were also evaluated. The size of the tumor was determined based on the maximum diameter either in a CC or MLO image. For quantitative data, we calculated the mean of two readers. For qualitative data, the final imaging features were confirmed when the two readers reached a consensus.

### Mammographic Texture Analysis

**Figure 2:**
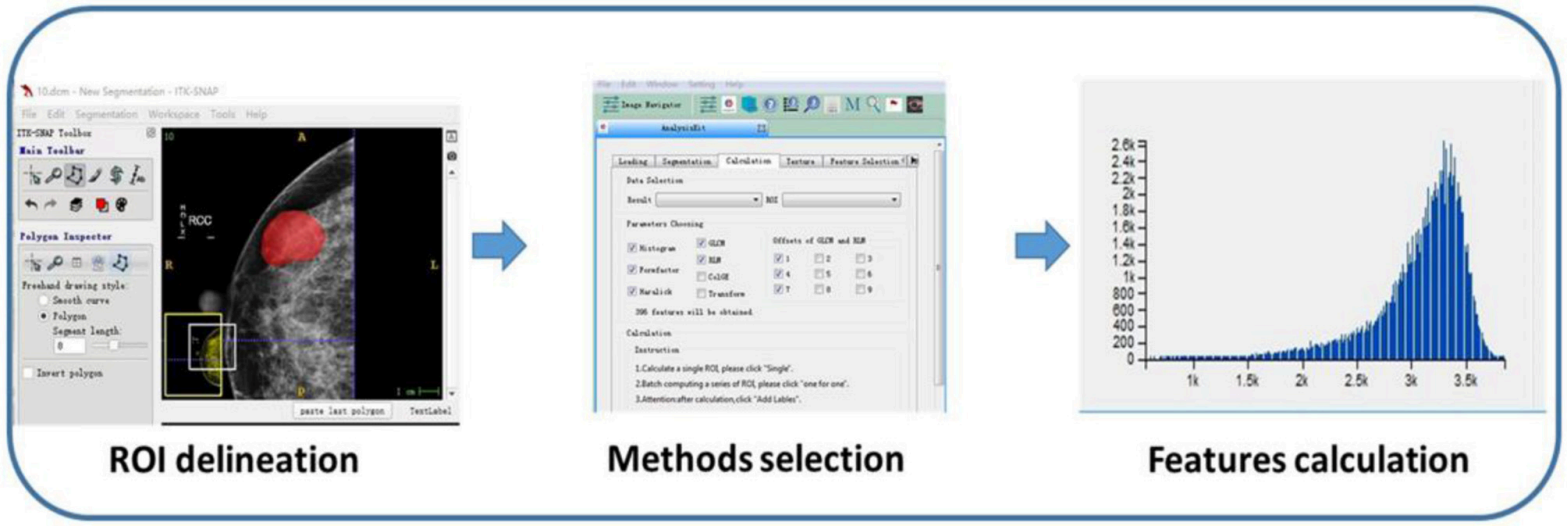
Flow diagram of texture features calculation.

Region of interests (ROIs) were drawn manually to delineate the lesions using ITK-SNAP software. Since PTs have envelopes and the display rate of a halo-ring is as high as 91.11%(41/45) in this study, we outline ROIs of tumors with a halo-ring as the boundary. All the dicom images and ROIs were individually transferred to the texture analysis software package (Artificial Intelligent Kit-A.K., GE Healthcare). Subsequently, texture features were automatically calculated by the A.K. software package. The texture analysis was performed twice for each lesion, and mean values of texture features were calculated. The procedure is shown in Figure 2. Three categories of statistical methods including Histogram, Gray Level Cooccurrence Matrices (GLCM), and run-length matrix (RLM) were used. A total of 435 texture features were extracted from each image in our study.

### Statistical Analysis

Statistical analyses were performed using IBM SPSS version 22.0 (IBM Corporation, New York). Quantitative data were displayed as mean ± SD. The Independent sample *t*-test and Mann-Whitney *U*-test was used for data with a normal and abnormal distribution, respectively. Categorical data were shown as a percentage and were analyzed using the Chi-square test or Fisher's exact test. Spearman correlation analysis and Logistic regression was used to show the relationship between texture features and tumor grade. *P* < 0.05 were considered statistically significant. The Receiver operating characteristic (ROC) curve was adopted to determine the diagnostic sensitivity and specificity of the Mammography and Mammographic texture analysis.

## Results

### Patients' Clinical Characteristics

The clinical characteristics of the 80 patients are summarized in Table 1. Each patient has only one lesion in the unilateral breast. All patients underwent surgery. There were 21 benign (26.25%), 38 borderline (47.50%), and 21 malignant tumors (26.25%). Fifteen of them underwent local excision, 52 underwent wide excision and 13 underwent mastectomy. Many PTs G2/G3 rapidly increased (diameter doubling) within half a year compared with PTs G1 (47.46 vs. 14.28%, *p* = 0.007). PTs G2/G3 were more likely to cause pain and skin changes compared to PTs G1 (*p* = 0.022). No significant differences were found in stiffness and mobility. Except for 19 lesions growing in the center or occupying the entire breast it was difficult to judge the origins, the location had no significance between these two groups.

**Table 1:** Clinical data of patients.

**Clinical data**	**Total *n* = 80**	**PTs G1 *n* = 21**	**PTs G2/G3 *n* = 59**	***p*-value**
**Age (years)**	46.58 ± 9.54	45.33 ± 7.91	47.02 ± 10.16	
**Growth speed**				0.007
Slowly increased	49	18	31	
Rapidly increased	31	3	28	
**Clinical manifestations**				0.022
Mass	48	17	31	
Mass with pain	19	4	15	
Mass with pain and skin change	13	0	13	
**Stiffness**				0.353
Hard	45	10	35	
Medium	35	11	24	
Soft	0	0	0	
**Mobility**				0.178
Well	39	11	28	
Not good enough	27	9	18	
Poor	14	1	13	
**Location**				0.088
Upper inner quadrant	11	1	10	
Lower inner quadrant	5	1	4	
Upper outer quadrant	37	11	26	
Lower outer quadrant	8	5	3	

### Mammography Findings

**Figure 3:**
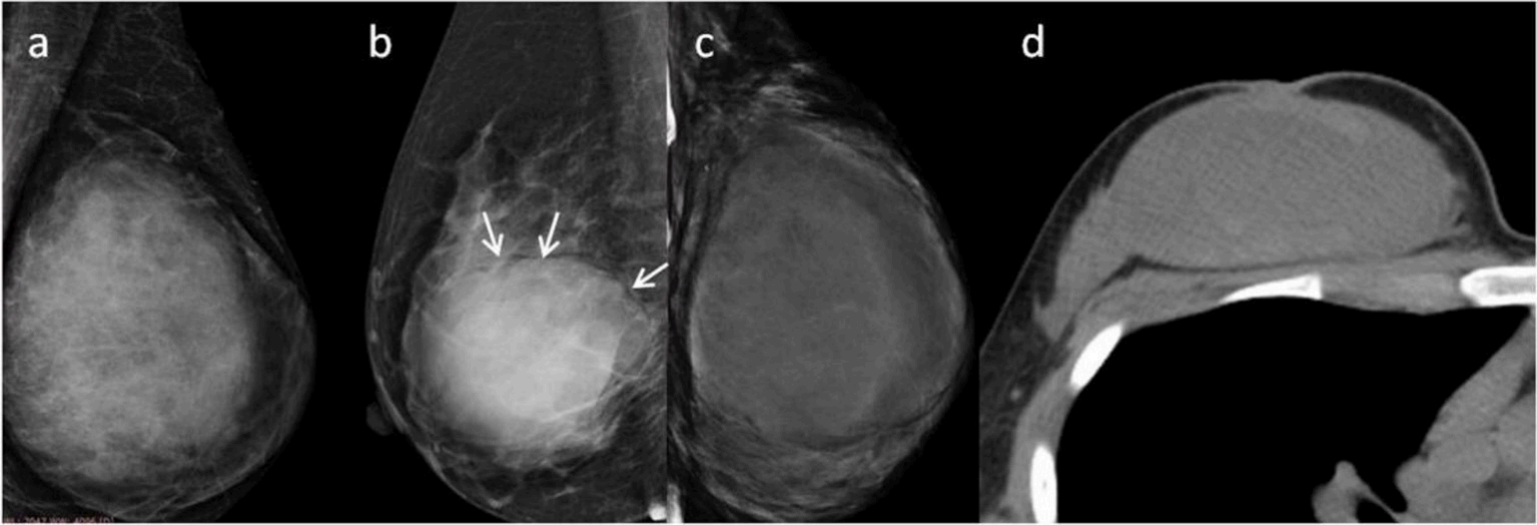
**(a)** Malignant Phyllodes tumor (PT) of left breast in a 55-year-old woman. A mediolateral oblique mammogram shows a well-defined isodensity mass with a diameter of 13 cm. **(b)** Malignant PT of right breast in a 47-year-old woman. Mammogram shows a well-defined high-density mass with a diameter of 9 cm. The mass is partially surrounded by a lucent halo (arrows). **(c,d)** Malignant PT of left breast in a 38-year-old woman. CT can show cystic changes within the tumor. They are all well-defined masses with large size.

**Figure 4:**
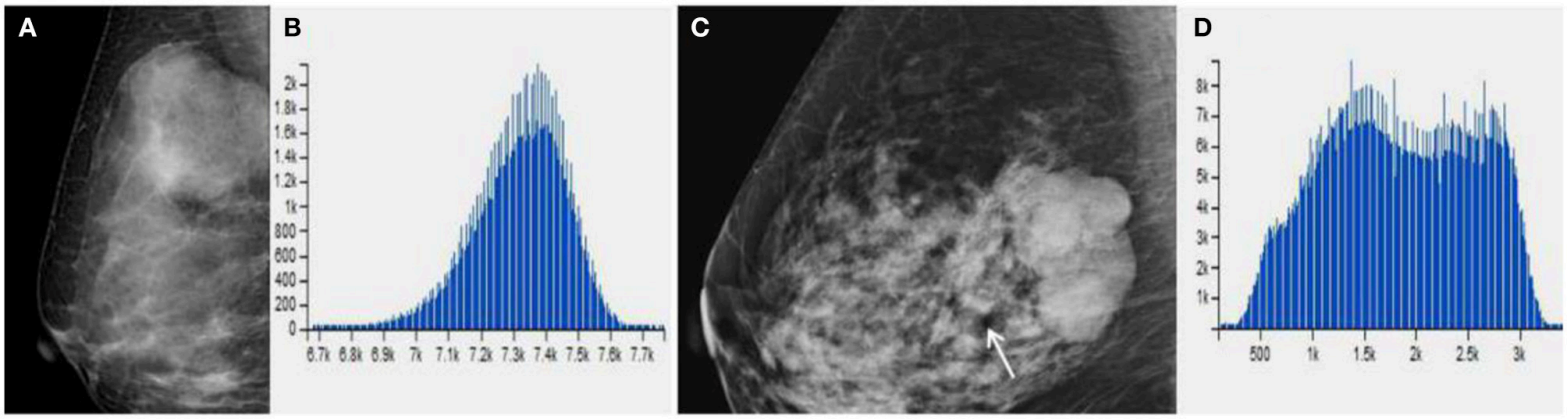
**(A)** Benign Phyllodes tumor (PT) of right breast in 49-year-old woman, mammogram shows an ovoid mass with a diameter of 4.5 cm. **(C)** Borderline PT with a diameter of 4.5 cm in 63-year-old woman. Mammogram shows a mass formed by multiple nodules. **(B,D)** The histogram of the texture parameters of the two lesions also show a marked difference.

**Figure 5:**
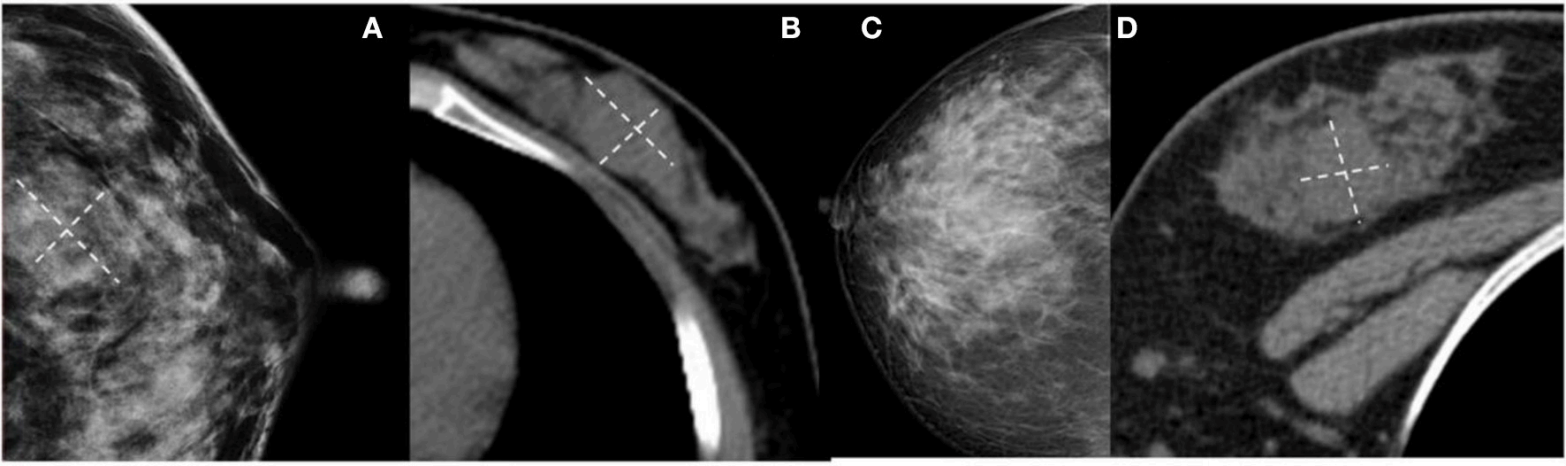
**(A,B)** Benign Phyllodes tumor (PT) of left breast in 55-year-old woman. Mammogram shows an ill-defined isodensity mass. However, CT can show the boundary clearly. **(C,D)** Benign PT of right breast in 34-year-old woman. The lesion is not visible on mammogram, but clearly visible on CT. They are all affected by the cover effect of mammography.

Subsequently, we evaluated the Mammographic findings of the 45 patients who met the study criteria. Their mean age was 48.2 ± 8.96. There were 14 benign (31.1%), 20 borderline (44.4%), and 11 malignant tumors (24.4%). Mammography findings of PTs are summarized in Table 2. Significant differences were found in tumor size, shape between G1 and G2/G3 PTs (*p* < 0.05). Larger size (*d* > 4.0 cm) were more common in G2/G3 PTs compared with PTs G1 (64.52 vs. 28.57%, *p* = 0.025) (Figure 3). PTs G2/G3 showed strong lobulation or multinodular confluence compared to the PTs G1 [20/31 (64.52) vs. 2/14 (14.29%), *p* = 0.004]. The lesions with strong lobulation or multinodular confluence showed a “multi-boundary sign” in MG because of the overlapped effect (Figure 4). Some low-grade PTs showed an ill-defined margin which was under the influence of the cover effect because of their small size and equal density to the surrounding gland (Figure 5). There were some limitations in the evaluation of PTs boundaries. There were no significant differences in density, homogeneity, the presence or absence of a halo ring, calcifications and fat between PTs G1 and PTs G2/G3. Similar results were observed for the indirect manifestations (Table 3).

**Table 2:** The mammography findings in phyllodes tumors (PTs) G1 and G2/G3.

**Mammographic findings**	**Total *n* = 45**	**PTs G1 *n* = 14**	**PTs G2/G3 *n* = 31**	***p*-value**
**Size (cm)**	5.54 ± 3.67	4.11 ± 2.55	6.19 ± 3.93	0.077
≤ 4	21	10	11	0.025
> 4	24	4	20	
**Shape**				0.004
Oval	10	6	4	
Weak lobulation	13	6	7	
Strong lobulation or multinodular confluent	22	2	20	
**Mass margin**				0.147
Well- defined	33	8	25	
Ill- defined	12	6	6	
**Density**				1.000
Hypodensity	1	0	1	
Isodensity	25	8	17	
Hyperdensity	19	6	13	
**Homogeneity**				0.725
Yes	33	11	22	
No	12	3	9	
**Halo sign**				0.082
Presence	41	11	30	
Absence	4	3	1	
**Calcifications**				0.578
Presence	4	2	2	
Absence	41	12	29	
**Fat**				1.000
Presence	1	0	1	
Absence	44	14	30	

**Table 3:** The indirect manifestations on mammography in Phyllodes tumors (PTs) G1 and G2/G3.

**Indirect mammography findings**	**Total *n* = 45**	**PTs G1 *n* = 14**	**PTs G2/G3 *n* = 31**	***p*-value**
**Breast composition categories of ACR**				0.889
a	2	1	1	
b	6	1	5	
c	30	10	20	
d	7	2	5	
**Skin thickening**				0.156
Presence	6	0	6	
Absence	39	14	25	
**Venectasia**				0.469
Presence	10	2	8	
Absence	35	12	23	
**Axillary lymphadenectasis**				0.530
Presence	2	1	1	
Absence	43	13	30	

**Figure 6:**
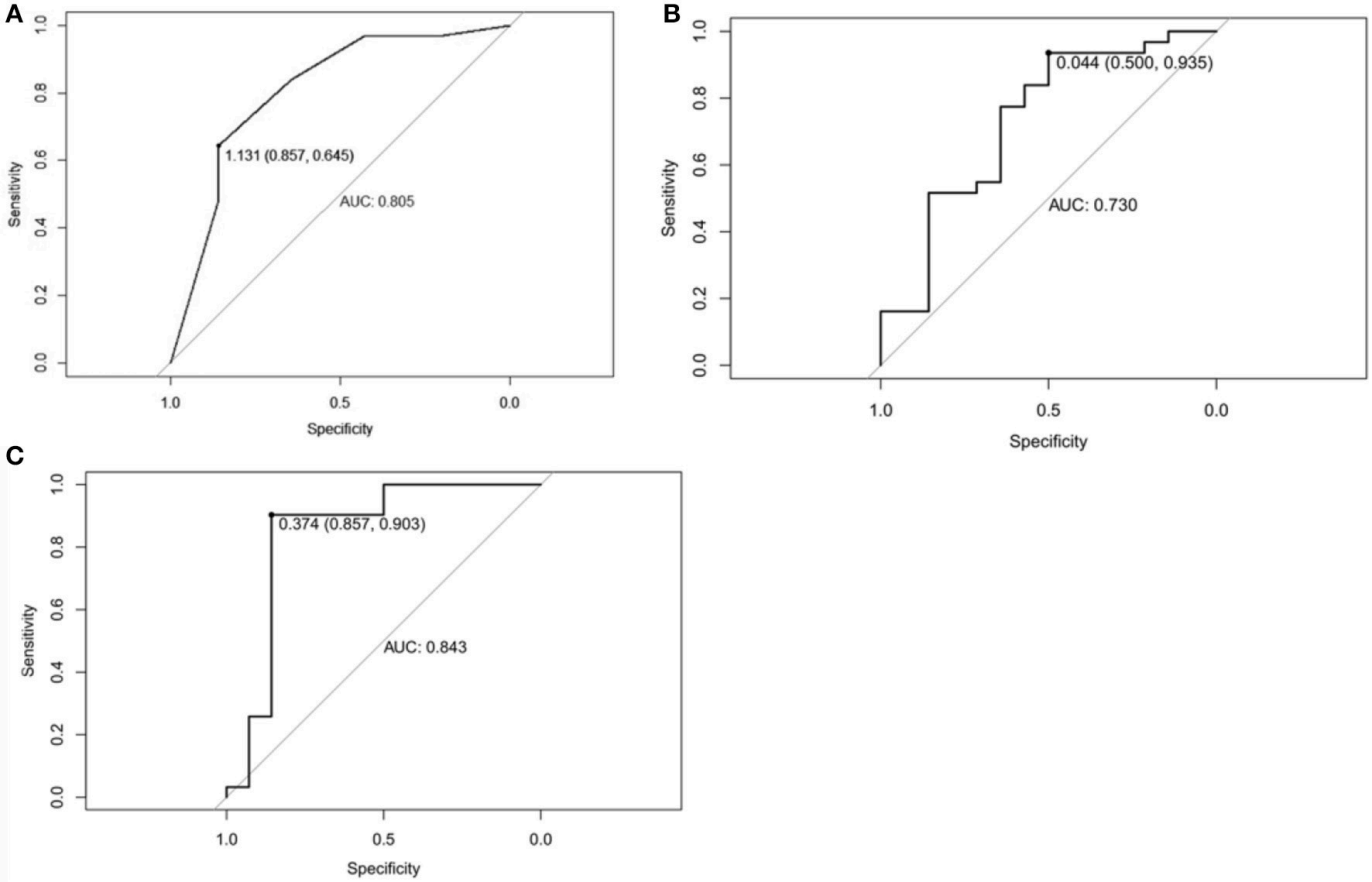
**(A)** Receiver operating characteristic curve of Mammographic findings in predicting Phyllodes tumor (PT) G2/G3 tumors. The area under curve was 0.805. **(B)** Receiver operating characteristic curve of texture features in predicting Phyllodes tumor (PT) G2/G3 tumors. The area under curve was 0.730. **(C)** Receiver operating characteristic curve of Mammographic findings + texture features in predicting PTs G2/G3 tumors. The area under curve was 0.843.

ROC curve was adopted to determine the diagnostic sensitivity and specificity of Mammography findings. AUC was 0.805 with 64.5% of sensitivity and 85.7% of specificity (Figure 6A).

### Mammographic Texture Analysis

**Figure 7:**
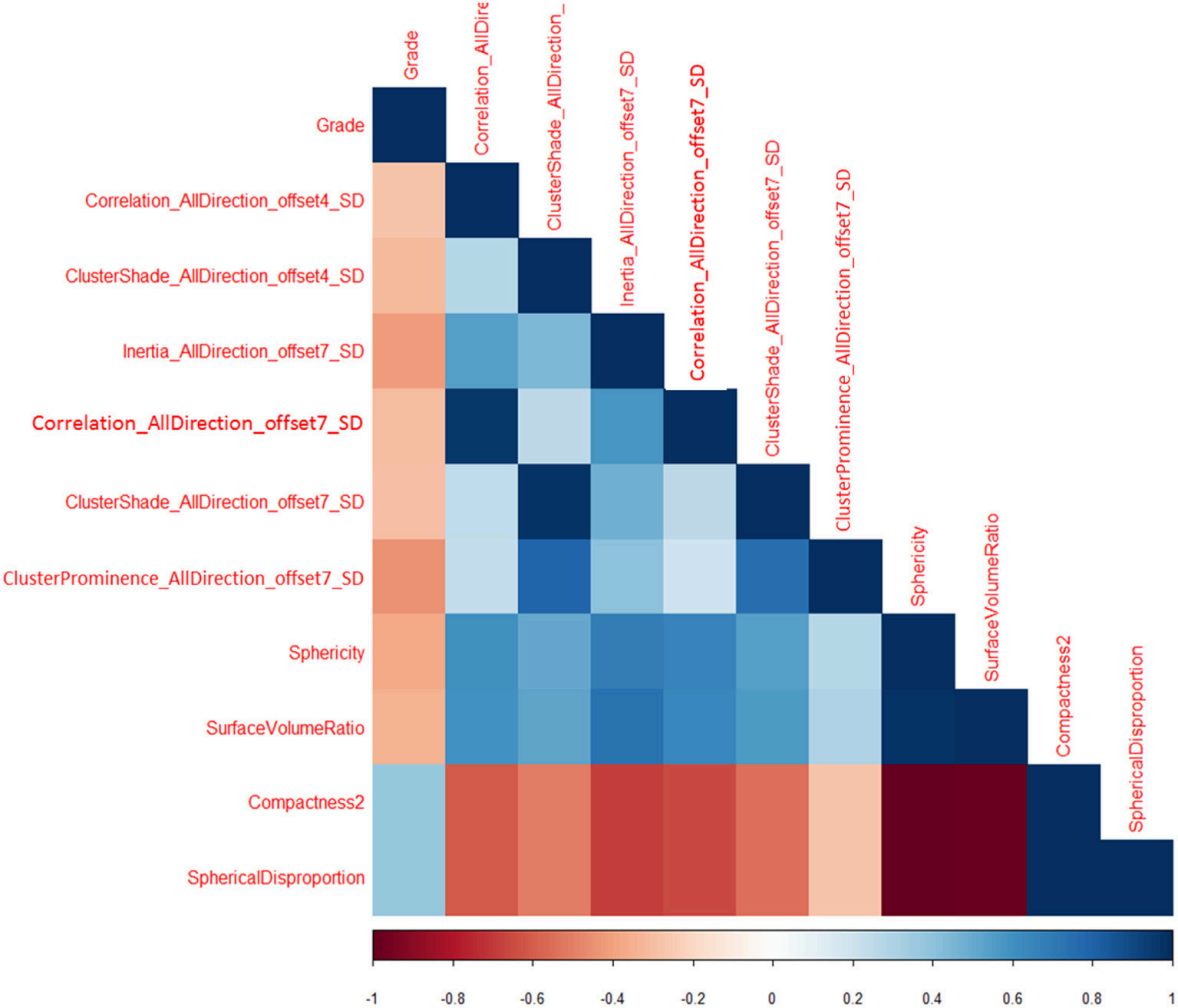
Correlation between texture parameters and tumor grading.

Total of 435 texture features were extracted from the mammographic images. Those texture features with significant differences between PTs G1 and PTs G2/G3 are shown in Table 4. Spearman correlation analysis also eliminated some parameters with strong a correlation (Figure 7). Finally, logistic regression showed that only two parameters were retained in our model. They were Correlation_AllDirection_offset7_SD and ClusterProminence_AllDirection_offset7_SD.

**Table 4:** Texture parameters in Phyllodes tumors (PTs) G1 and G2/G3.

**Mammographic texture analysis**	**Total *n* = 45**	**PTs G1 *n* = 14**	**PTs G2/G3 *n* = 31**	***p*-value**
Sphericity	0.21 ± 0.06	0.24 ± 0.06	0.20 ± 0.06	0.021
Surface volume ratio	2146.45 ± 73.01	2174.47 ± 74.15	2133.79 ± 68.84	0.044
Compactness2	134.35 ± 74.93	105.36 ± 51.65	147.44 ± 79.96	0.042
Spherical disproportion	5.27 ± 1.87	4.51 ± 1.39	5.61 ± 1.95	0.035
Correlation_AllDirection_offset4_SD( × 10^−6^)	6.56 ± 5.45	8.63 ± 6.03	5.62 ± 4.99	0.043
Correlation_AllDirection_offset7_SD( × 10^−5^)	1.20 ± 1.08	1.77 ± 1.34	9.49 ± 8.43	0.008
ClusterShade_AllDirection_offset4_SD ( × 10^3^)	8.86 ± 7.83	11.86 ± 10.25	7.50 ± 5.90	0.039
ClusterShade_AllDirection_offset7_SD ( × 10^3^)	13.77 ± 11.84	18.93 ± 16.21	11.44 ± 8.58	0.024
ClusterProminence_AllDirection_offset7_SD( × 10^6^)	5.36 ± 4.31	7.03 ± 6.24	4.598217.58 ± 2.90	0.040
Inertia_AllDirection_offset7_SD	58.16 ± 38.71	72.87 ± 49.94	51.51 ± 31.16	0.043

#### Parameter 1:Correlation_AllDirection_offset7_SD

Correlation measures the similarity of the gray levels in neighboring pixels. Correlation_AllDirection_offset7_SD is one of the 18 parameters related to the Correlation in AK Software.

$$\begin{array}{l} \text{Formula}:-\underset{i,j}{\sum }\frac{\left(i-\mu \right)\left(j-\mu \right)\text{g}\left(i,j\right)}{\sigma 2} \end{array}$$

#### Parameter 2:ClusterProminence_AllDirection_offset7_SD

Cluster Prominence is a measure of a symmetry of a given distribution. High values of this feature indicate that the symmetry of the image is low, in medical imaging low values of cluster prominence represent a smaller peak for the image gray level value and usually the gray level difference between the forms is small.

$$\begin{array}{l} \text{Formula}:\underset{i,j}{\sum }\left(\left(i-\mu \right)+\left(i+\mu \right)\right)4g\left(i,j\right) \end{array}$$

The texture features were associated with tumor grade (OR = 0.465, 95%CI:0.231–0.936; OR = 0.042, 95CI:0.193–0.969, respectively).

ROC curve was adopted to determine the diagnostic sensitivity and specificity of Mammographic texture analysis. The AUC was 0.730. When the cut off value was 0.044, the sensitivity was 93.5%, and the specificity was 50% (Figure 6B).

Subsequently, ROC curve was also adopted to determine the diagnostic sensitivity and specificity of Mammography findings + texture features. The AUC was 0.843 with 90.3% sensitivity and 85.7% specificity for predicting PTs G2/G3 tumors (Figure 6C).

Finally, we randomly selected 30 samples for internal validation, including nine benign (30%), 13 borderline (43.33%), and eight malignant tumors (26.67%). The AUC was 0.862 (85.7% sensitivity and 77.8% specificity). The verification results are similar to those of previous studies, which prove that the model is relatively stable.

## Discussion

Previous studies have indicated that imaging approaches are useful in differentiating PTs G1 from PTs G2/G3. In the present study, we evaluated the role of texture features in grading PTs. Our data indicated that texture features are useful in grading PTs. Moreover, our data indicates that texture analysis can improve the diagnostic performance in differentiating PTs G1 and PTs G2/G3.

Surgical methods are associated with the grades of PTs. The preoperative differentiation would be especially useful for surgery planning. A fine-needle biopsy is an accurate method used in the diagnosis of PTs but cannot be used for classification, because of inadequate cytologic samples and the heterogeneous nature of the tissue composition(10, 11). It would be helpful to evaluate the PTs grades by using imaging approaches. However, the radiologic studies in PTs grading are very few because of the low incidence. Previous US, MG, and MRI studies indicated that a larger tumor size and irregular tumor shape are more common in higher grades of tumors than in lower grade tumors (9–15). Our data is consistent with those previous findings, and we found that the multinodular confluent was characteristic imaging manifestation of PTs G2/G3. This is related to the degree of leaf-like growth in histology (2). An irregular cyst wall in an MRI, a tumor signal intensity lower than or equal to normal tissue on T2-weighted images and a low apparent diffusion coefficient (ADC) are all significantly correlated with the histologic grade. T1 weighted imaging signal in the G2/G3 PTs was higher than that in the PTs G1 (12).

Recently, texture analysis has been widely used to evaluate tumor heterogeneity. Texture parameters, such as entropy and kurtosis, show good performance in differentiating benign from malignant tumors (16, 17). Several studies also indicate that texture features are good predictors of tumor grades (18, 19). However, few studies have shown the role of texture features in PTs grading. We were the first one to use the method of mammographic texture analysis to grade the PTs up to now. Significant differences were found in 10 texture features and Correlation_AllDirection_offset7_SD and ClusterProminence_AllDirection_offset7_SD were the independent factors in identifying PTs G1 from PTs G2/G3. In addition, our data also indicated that Mammography can obtain good specificity but poor sensitivity, while texture analysis can obtain high sensitivity but poor specificity in differentiation. Interestingly, the combination of the two approaches can obtain both high sensitivity and specificity. Texture analysis can effectively improve the efficacy of mammography for PTs classification.

There are also several limitations in our study. First, since a mammography is a two-dimensional structural image, the recognition of functional, and three-dimensional structural images is absent, and texture analysis based on mammography may lose a lot of information. Second, as a retrospective study, selection bias cannot be avoided. Third, it is inevitable that the number of patients in this study is small for texture analysis study. There are two main reasons for the small number of cases: (1) The incidence of PTs is low, which only accounts for 1% of breast tumors. It is relatively difficult to collect cases for this. Second, texture analysis research requires a high consistency of Imaging equipment and parameters, in order to ensure the accuracy of texture analysis, some cases have to be excluded from the study. Because of the relatively small sample size, all cases were included for texture feature extraction. Then we performed internal validation to verify the results, aiming to improve the accuracy of the test set as much as possible under existing conditions. Finally, we compared the texture analysis results obtained in this study, with previous literature, and found that the two independent parameters we screened had been reported to have clear statistical significance in the benign and malignant differentiation of breast calcifications and evaluation of chemotherapy efficacy (20, 21), which further supported the credibility of the results of this study. In the future, we will expand the sample size to further improve the accuracy and repeatability of the study.

In conclusion, our data indicates that texture analysis based on Mammography has the potential to differentiate PTs G2/G3 from PTs G1. Combining Mammography and texture features can provide optimal predictions in the classification of PTs in mammography.

## Data Availability

All datasets generated for this study are included in the manuscript and/or the supplementary files.

## Contribution to the Field Statement

Phyllodes tumors (PTs) are rare breast fibroepithelial neoplasms that account for <1% of all breast tumors. PTs are classified as benign, borderline, and malignant. The preoperative differentiation between PTs G1 and G2/G3 would be especially useful for surgery planning. Wide excision or mastectomy is usually performed in PTs Grade2 (G2)/G3. A fine-needle biopsy should not be used for PTs grading because of the inadequate cytologic samples and the heterogeneous nature of the tissue composition. MRI may be a useful imaging approach but has so much contraindication as well as being costly. The MG and US showed limited potential in predicating PTs grades, however, our study first used the method of mammography combined with mammographic texture analysis to grade the PTs. The area under the curve (AUC) of imaging-based diagnosis, texture-based diagnosis and the combination of the two approaches were 0.805(64.5% sensitivity and 85.7% specificity), 0.730 (93.5% sensitivity and 50% specificity), and 0.843 (90.3% sensitivity and 85.7% specificity). Texture analysis can effectively improve the efficacy of mammography for PTs classification.

## Author Contributions

WC and ZW: guarantor of integrity of the entire study. ZW and XC: study concepts and design. CW, WC, and CC: clinical studies. CW, SR, and SD: statistical analysis. WC, XC and LJ: manuscript editing.

### Conflict of Interest Statement

The authors declare that the research was conducted in the absence of any commercial or financial relationships that could be construed as a potential conflict of interest.

## Abbreviations

G1: Grade 1
G2/G3: Grade 2/Grade 3
PTs: Phyllodes tumors
MG: mammography
ROC: Receiver operating characteristic
US: ultrasound
AUC: Area under the curve
MRI: magnetic resonance imaging
CC: craniocaudal
MLO: mediolateral oblique
PACS: Picture Archiving and Communication Systems
ACR: American College of Radiology
ROIs: Region of interests
GLCM: Gray Level Co-occurrence Matrices
RLM: run-length matrix
AI: artificial intelligent
ADC: apparent diffusion coefficient.
